# Cerium and europium doped TiO_2_ thin films deposited by a sol-gel dip-coating process: characterization and photocatalytic activity toward dye degradation

**DOI:** 10.55730/1300-0527.3317

**Published:** 2021-12-01

**Authors:** Dalanda HAMDI, Lobna MANSOURI, Varsha SRIVASTAVA, Mika SILLANPAA, Latifa BOUSSELMI

**Affiliations:** 1 Laboratory of Wastewater and Environment, Center for Water Research and Technologies of BorjCedria Tunisia (CERTE), Technopark Borj Cedria, Soliman, Tunisia; 2 University of Gabes, National school of engineers of Gabes, Gabes, Tunisia; 3 Research Unit of Sustainable Chemistry, Faculty of Technology, University of Oulu, Oulu, Finland; 4 Environmental Engineering and Management Research Group, Ton Duc Thang University, Ho Chi Minh City, Vietnam; 5 Faculty of Environment and Labour Safety, Ton Duc Thang University, Ho Chi Minh City, Vietnam

**Keywords:** TiO_2_, cerium, europium, sol-gel, dip coating, silicon, photodegradation

## Abstract

Cerium (Ce) and europium (Eu)-doped TiO_2_ thin films were obtained by sol-gel dip-coating technique. SEM micrographs showed that the surfaces are covered by agglomerated particles due to the repeating coating process. XRD patterns showed the presence of TiO_2_ anatase phase. Raman spectra revealed that the peaks recorded at 146 cm^−1^(E_g_) and 397 cm^−1^(B_1g_) were related to the anatase phase. EIS measurements proved that Ce-TiO_2_ (1wt%) and Eu-TiO_2_ (0.1wt%) photocatalysts possessed a lower electron transfer resistance than pure TiO_2_, which can lead to effective separation of electron/ hole pairs during the photoreactions. The photoactivity of Ce and Eu-doped TiO_2_ was investigated by the degradation of amido black10B dye (AB) under UV excitation and varying the initial pH and concentrations. It was found that Eu-TiO_2_ (0.1wt%) exhibited higher photocatalytic activity, reaching a first-order reaction rate of *k_app_* (0.036min^−1^), t_1/2_ was around 12 min and AB removal was 98.94%, under optimal pH of 3.5 and AB concentration of 10ppm compared to (t_1/2_= 45 min, t_1/2_=30 min), (*k_app_*= 0.022 min^−1^, *k_app_*=0.026min^−1^) and AB removal (94.78%, 96.44%), respectively for pure TiO_2_ and Ce-TiO_2_ (1wt%). Further increase in Eu/Ce amount up to optimal concentration (1wt% Ce and 0.1wt% Eu) led to a decrease in the AB removal. The mineralization of AB using Eu-TiO_2_ photocatalyst was confirmed by HPLC analysis.

## 1. Introduction

TiO_2_ catalyst has been proven to be the widely used material in several applications [1,2]. Due to its multi-faceted functional properties, TiO_2_ is known as a promising photocatalyst material generally used in photocatalytic reactions for water treatment process [3]. Besides its strong mechanical properties, TiO_2_ has received considerable attention as it is inexpensive, chemically stable, nontoxic, and with great oxidation ability [4]. However, anatase TiO_2_ is a material with wide band gap (Eg = 3.23eV) that exhibits recombination of electron/hole pairs [5]. Thereby, several researchers have modified TiO_2_ nanomaterials using rare earth elements (RE) such as Ce, La, and Er to enhance the photocatalytic activity under UV irradiation [6,7]. Among them, Ce ions have strong absorption ability and showed efficient optical properties to improve the photocatalytic efficiency of TiO_2_ photocatalyst [8]. In fact, Ce ions can enhance the efficiency of the photodegradation via inhibiting the recombination rate of photogenerated electron/ hole pairs by acting as electron traps [9]. Furthermore, Ce ions have been reported to shift the band edge in TiO_2_ to the visible region and improve the redox potential of the photo-generated radicals [7,10]. On the other hand, Eu ions doping has attracted much attention in photocatalytic process due to its higher photocatalytic activity than pure TiO_2_ in the degradation of organic pollutants [11]. Thereby, recent studies have reported the photocatalytic enhancement of RE-doped TiO_2_ thin films [9,12,13,14,15,16,17,18,19]. TiO_2_ photocatalyst can be used in slurry or immobilized systems [20]. However, in a slurry system the separation step of the catalyst particles constitutes a major drawback, and it is needed to avoid as it is costly, unless it remains mandatory [21]. Thereby, the photocatalytic oxidation using an immobilized TiO_2_ photocatalyst is more recommended [22]. Silicon (Si) is a semiconductor with a narrow indirect band gap (1.12 eV) [23]. p-Si was used as photocatalyst substrate [24], because its abundant and eco-friendly [25]. Therefore, Si is an encouraging material for making eco-friendly and visible-light active photocatalyst-based heterostructures. p-Si used as substrate was reported to show an enhancement in the photocatalytic activity of n-photocatalyst compared to the glass substrate [26]. This enhancement in photocatalytic activity can be explained by the contribution of the inner electric field of the p-Si/ n-TiO_2_ heterojunction to the separation of photogenerated electron/ hole pairs. Thereby, when photons of the p/ n heterojunction are irradiated with energies equal or higher than the band gaps of n-TiO_2_ and p-Si, electron/ hole pairs are generated. Due to the action of the inner electric field, the photogenerated electrons can be injected from the conduction band of p-Si to that of n-TiO_2_. In the valence band, the photogenerated holes migrate in the reverse direction [27,28]. Subsequently, the photogenerated electrons in the n-TiO_2_ conduction band can be trapped by the adsorbed O_2_ molecules to produce superoxide radical anions, and the photogenerated holes in p-Si valence band can be scavenged by the H_2_O molecules to produce •OH that are responsible for degradation of organic molecules. Therefore, the photogenerated electrons and holes are efficiently separated and the recombination of electron/ hole pairs is suppressed, and the photocatalytic activity is enhanced. For these reasons, various methods have reported the synthesis of TiO_2_ thin films such as: chemical vapor deposition (CVD) [29,30], atomic layer deposition [31,32,33], electrochemical anodization [34] and sol-gel process [35,36]. Particularly, the sol-gel technique involves thin film depositions with a large specific surface area, high purity and mechanical resistance, good crystallinity, chemical durability, and controlled conditions of morphology, composition, and thickness [37,38]. Among sol-gel process, the dip-coating technique is used for immobilization purposes in a wide range of applications [39,40]. The dip-coating process is an economically feasible technique. It can be carried out at room temperature, and applied to a wide variety of substrates with large surfaces and various form [41]. The dip-coating process can produce films with high uniformity and a thickness ranging from nanometers to 200 nm for oxide films [42]. The good uniformity of the thin films obtained by dip-coating technique can be reached due to the layer-by-layer growth during each dip process of the substrate in the precursor solution [43]. The sol-gel dip-coating technique is essentially based on mechanisms of hydrolysis and polycondensation of titanium alkoxides mixed with alcohol and catalytic agents [35,44].

In the present study, Ce or Eu doped-TiO_2_ thin films are coated on both sides, by the sol-gel dip coating process, on p-silicon wafers (p-Si). The effect of dopant ions on the structural, morphological properties and photocatalytic activity under UV irradiation are discussed in details. Amido black 10B dye was selected, as a model pollutant, to test the photocatalytic activity of this new synthesized materials, under different operational conditions (initial pH value (3.5, 5.5, 7, and 9), dye concentration (10, 20, 30, and 40 ppm). The photo-electrochemical behavior of the pure and doped TiO_2_ electrodes was carried out by the measurements of the electrochemical impedance spectroscopy (EIS) in the dark before and after UV irradiation in alkaline medium.

## 2. Experimental

### 2.1. Reagents

Titanium (IV) isopropoxide (C_12_H_28_O_4_Ti; CAS Number 546-68-9; purity: 97 %), acetic acid (≥99.5%), Amido Black 10B (C_22_H_14_N_6_Na_2_O_9_S_2_), and absolute ethanol (99.98%) were used without purification and were purchased from Sigma Aldrich. Cerium (III) nitrate hexahydrate (99% trace metal basis) and europium (III) nitrate pentahydrate (99.9%) were obtained from Aldrich Chemistry. Hydrofluoric acid (40% purity) was acquired from Emsure. Sodium hydroxide was purchased from Sigma Aldrich. All solutions were prepared using ultrapure water (18.2 MΩ) produced in a Direct-Q millipore system. Silicon wafers (CAS Number 7440-21-3) p-type (100) were provided by Si-Mat silicon materials, Germany.

### 2.2. Synthesis and coating of Ce and Eu doped TiO_2_ thin films on p-silicon wafers

In the present study, pure and doped TiO_2_ thin films were cast by the dip-coating process using of titanium (IV) isopropoxide as a precursor of TiO_2_. Pure, Ce, and Eu doped TiO_2_ thin films were coated, on p-silicon wafers on both sides, using a HOLMARCs dip coating unit with the infrared dryer (HO-TH-02B). Firstly, 18 mL of titanium (IV) isopropoxide was dissolved in 100 mL of ethanol and stirred for 30 min, then 3 mL of acetic acid was added dropwise and the mixture was stirred again for 5 h. When required, different atomic weight percentages of Ce (NO_3_)_3_ (Ce: 0.1, 0.5, 1, 3, and 5wt%) or EuH_10_N_3_O_14_ (Eu: 0.1, 0.5, and 1wt%) were added to the mixture, respectively, for the synthesis of Ce-TiO_2_ and Eu-TiO_2_ films. The substrates were immersed in the titanium precursor solution for 2 min and withdrew at a constant speed of 5mm.s^−1^ to deposit one layer of the film. After each dip process, the samples were automatically dried at 100 °C for 10 min, to evaporate the solvent. The process of dip-drying was repeated five times. Then, the prepared thin films were annealed at 500 ^o^C by heating at a defined rate, for 2h. It is necessary to report that prior to deposition, the rectangular p-Si substrates (10.5cm^2^) were cleaned with hydrofluoric acid and ethanol, then rinsed with deionised water and finally dried in order to remove native oxide.

### 2.3. Analytical methods

#### -Structure and morphology

The morphological properties of pure TiO_2_, Ce-TiO_2,_ and Eu-TiO_2_ thin films were studied by means of SEM-EDS microscope (Hitachi S-4800 Ultra-High Resolution Scanning Electron Microscope High-Resolution Field-Emission scanning electron microscope (SEM)) having a resolution of 1nm. The crystalline structure analysis of the samples was studied using X-ray diffraction radiation (XRD) analysis using XRD PANalytical Empyrean. Raman spectra were investigated on a JYT64000 Raman spectrometer Horiba Jobin Yvon, Labram HR at green laser (514.53 nm).

#### -Photoelectrochemical and electrochemical measurements

They were performed in 0.1M NaOH at room temperature using three-electrode cell quartz (100 mL), where the elaborated materials, served as working electrodes, a platinium eletrode served as counter-electrode. All potentials were measured and referred to a saturated calomel reference electrode (SCE). Photoelectrochemical characterization were performed using a VoltaLab 40 PGZ301 potentiostat (Radiometer Analytical), connected with a computer that uses VoltaMaster 4.0 software for data acquisition. EIS measurements were carriedout with 10 mV applied sinusoidal AC perturbation over the frequency range of 0.125–10^5^ Hz. Photopotential (Eocp) measurements of the electrodes were carried out when no bias is applied to the photoelectrode, during on/off cycle of UV irradiation, provided by a high pressure mercury lamp (Cathodeon HPK 27.5 mW; λ = 365.5nm), exposed to one-coated side of the eletrodes. Data were recorded and fitted with Z-Simpwin 3.2 software. Photoelectrochemical measurements were performed in NaOH(0.1M) at room temperature using three-electrode quartz (100 mL), where the Ce-TiO_2_ (1wt%) and Eu-TiO_2_ (0.1wt%) (obtained under optimised conditions) and pure TiO_2_ photocatalyst, served as working electrodes. All potentials were measured and referred to a saturated calomel reference electrode (SCE). Photoelectrochemical characterization was performed using a VoltaLab 40 PGZ301 potentiostat (Radiometer Analytical), with VoltaMaster 4.0 software for data acquisition.

#### -Technical analysis

The HPLC spectra were recorded on an Agilent 1260 Infinity II liquid chromatography system with diode array detector (DAD, Agilent G1315D) set as 254nm. The chromatographic conditions were: mobile phase 60%v methanol: 40%v water, flow rate 1mL.min^−1^, injection volume 20 μL, chromatographic column LC18 column Zorbax ecli pse XDB (250×4.6 mm). UV-Visible spectra and analytical determination of AB remaining in solution were done calorimetrically (620nm) using UV-Vis spectrophotometer (Perken Elmer Lampda 450) and HPLC system with diode array detector (DAD, Agilent G1315D) set as 254nm.

### 2.4. Photodegradation experiments

The photocatalytic performance of the synthesized Ce-TiO_2_ and Eu- TiO_2_ thin films was evaluated by studying the discoloration of amido black dye solution as a model pollutant under UV light. The photodegradation experiments were carried out in a quartz beaker, equipped with a magnetic stirring bar. The UV irradiation was provided by two replacement tubes (HITACHI, shortwave 254nm) placed on parallel, in a way to effectively illuminate both coated surface sides. The synthesized Ce-TiO_2_ and Eu- TiO_2_ supported on silicon wafers were immersed in 50 mL of AB dye aqueous solution. The distance between the AB dye solutions and the UV light source was fixed to 10 cm, from both sides. During the different photocatalytic experiments, several operational parameters (AB concentration, pH, and type of the used catalyst), were varied to study their effects on pollutant degradation. The initial pH of AB aqueous solutions was adjusted prior to each experiment by NaOH or HCl solutions (0.1M).

## 3. Results and discussion

### 3.1. SEM-EDS analysis

**Figure 1:**
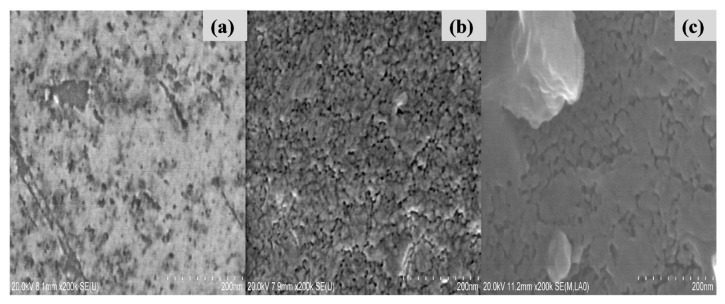
SEM analysis of (a) Pure-TiO_2_, (b) Ce-doped TiO_2_, and (c) Eu-doped TiO_2_ thin films.

Figure 1 shows examples of SEM patterns (top views) of the undoped and Ce or Eu- doped TiO_2_ films deposited on silicon wafers by sol-gel dip-coating technique (optimal photodegradation results). Figure 1 shows SEM images of annealed pure and doped-TiO_2_ thin films. The pure TiO_2_ sample seems to have a smooth surface without any cracks (Figure 1a). After Ce and Eu doping, the surfaces showed agglomerated particles for Ce-TiO_2_ and Eu-TiO_2_ samples. Thus, despite the absence of cracks and fissures as clearly shown by SEM analysis (Figure 1b and 1c), there is no doubt that the coating process repeated 5 times affected the agglomeration of the particles after adding dopant ions. In fact, the addition of Ce and Eu ions generated oxygen vacancies, as confirmed by Raman analysis (shift in E_g_ mode to higher wavenumber). The larger ionic radius of Ce^3+^ (0.111 nm) and Eu^3+^(0.095 nm) compared to Ti^4+^(0.068 nm) ions, make the Ce^3+^/ Eu^3+^ ions unable to substitute Ti^4+^ in the TiO_2_ lattice. The oxygen vacancy defects in case of Ce/Eu-doping generate a charge decompensation favoring the agglomeration process [45].

The average film thickness of the layers obtained from the cross-section (not shown here) is illustrated in Table 1. It is about 6.4 μm for pure TiO_2_ thin film. However, doping TiO_2_ thin film with Ce led to a decrease in film thickness up to 4.5, 4.1, 2.7, 1.4μm, respectively for (Ce: 0.1, 0.5, 1, 3 wt%), then increased again to 3.7 μm for Ce (5wt%). However, when TiO_2_ thin film is doped with Eu, the film thickness decreased up to 2.7 μm and 1.4μm, respectively for (Eu: 0.1 and 0.5 wt%), then increased again to 2.7 μm for Eu (1wt%) (Table 1). This behavior is linked to the fact that increasing gradually dopant concentration in the solution retards the growth of TiO_2_ thin film [46]. According to these results, the film thickness of pure TiO_2_ thin film prepared by sol-gel dip-coating technique in the present study is higher (6.4 μm) than that prepared by sol-gel spin-coating technique as reported by [47] (3.8 μm) in the same conditions of sol precursor solution and after five times coating. In fact, dip-coating technique requires the immersion of a substrate in titanium precursor in a volume solution of 50 mL and the spin coating technique requires only the use of a few drops of the prepared sol precursor. Thereby, TiO_2_ thin film thickness is expected to be higher than that prepared by sol-gel spin-coating technique.

**Figure 2:**
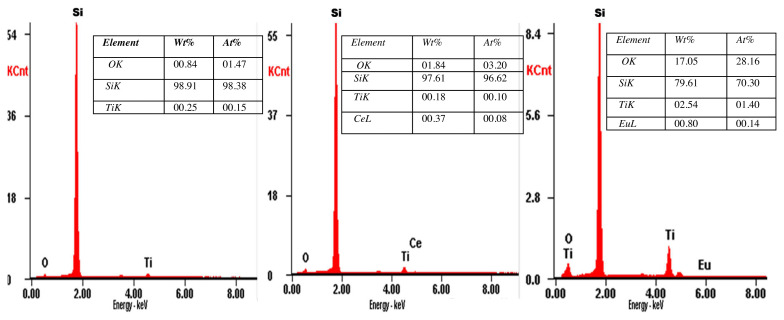
EDS spectra of (a) Pure-TiO_2_ thin films (b) Ce-doped TiO_2_ thin films (1wt%),(c) Eu- doped TiO_2_ thin films (0.1wt%).

The Energy Dispersive X-ray Spectroscopy (EDS) analysis reveals the presence of the Ti, O, Ce, and Eu peaks (Figure 2). During the doping process, Ce and Eu substitute Ti in the TiO lattice which is confirmed by the increase in Ce/ Ti atomic ratio in Ce-TiO_2_ thin films from 0 to up to 2.2 as the Ce amount in the precursor solution increased from 0 to 5wt% (Figure 2). Likewise, Eu/Ti atomic ratio increased from 0 to 0.35 as the Eu amount increased from 0 up to 1wt% (Figure 2).

### 3.2. XRD analysis

**Figure 3:**
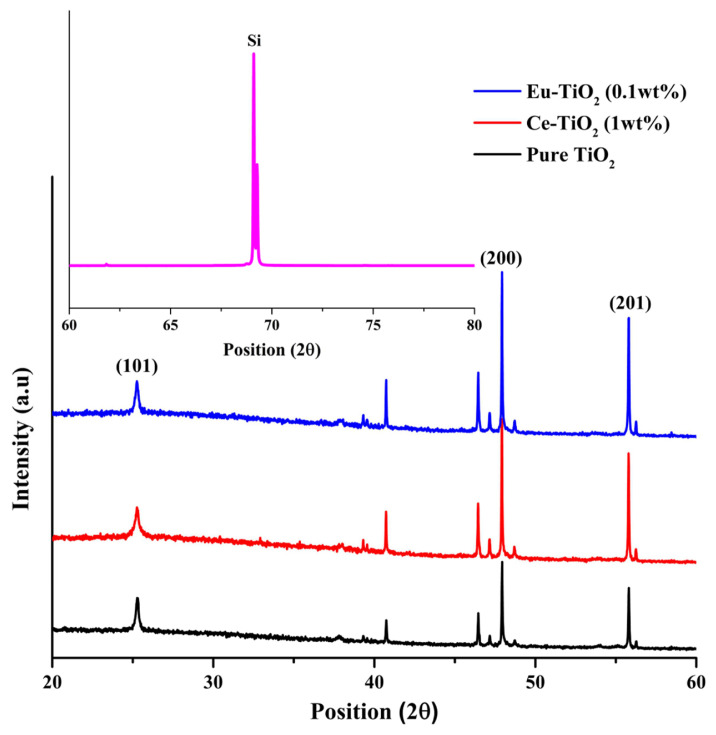
XRD patterns of Pure-TiO_2_, Ce- doped TiO_2_ (1wt%) and Eu- doped TiO_2_(0.1wt%) thin films annealed at 500 °C. Inset figure (silicon substrate), A=anatase, R=rutile.

The XRD patterns of the deposited pure- TiO_2_, Ce-doped TiO_2,_ and Eu-doped TiO_2_ thin films elaborated by dip coating and annealed at 500 °C, are illustrated in Figure 3. The obtained X-rays diffraction patterns exhibited mainly the anatase phase (JCPDS No. 21-1272) [48]. All the films showed the anatase predominant peaks having (101), (200) and (201) as orientations, and were observed, respectively at 2θ = 25°, 2θ = 48° and 2θ = 56° (Figure 3). Furthermore, the XRD patterns (Figure 3) show also another dominant peak at 2θ = 68°, which corresponds to p-Si substrate [47,49]. XRD patterns showed also a rutile peak having (111) orientation and observed at 2θ = 41° [50]. The calculated parameters of (101) crystal plane of the first anatase diffraction peak estimated from the Debye–Scherrer equation [51] of pure and Ce-TiO_2_ thin films are defined and reported in Table 2.

The crystallite size of TiO_2_ did not change when low doping amounts were introduced. The crystalline sizes are 27.70 nm and 27.69nm, respectively for Ce-TiO_2_ (1wt%) and Eu-TiO_2_ (0.1wt%) compared to 27.71 nm for pure TiO_2_. This behavior is confirmed by the stable anatase peak intensity after doping with Ce (1wt%) and Eu(0.1wt%), which revealed that the crystallinity of TiO_2_ lattice was not damaged after doping at a low amount (Figure 3). However, on Ce doping at a high amount, the anatase grain growth is hindered and the crystallite size decreased to 20.88 nm. This result is in agreement with the literature [52], Ce ions doped into host lattice leads to the creation of imperfection in the crystal structure. The crystal lattice expansion reduces the growth of anatase and thereby, the crystallite size was decreased [53].

### 3.3. Raman analysis

**Figure 4:**
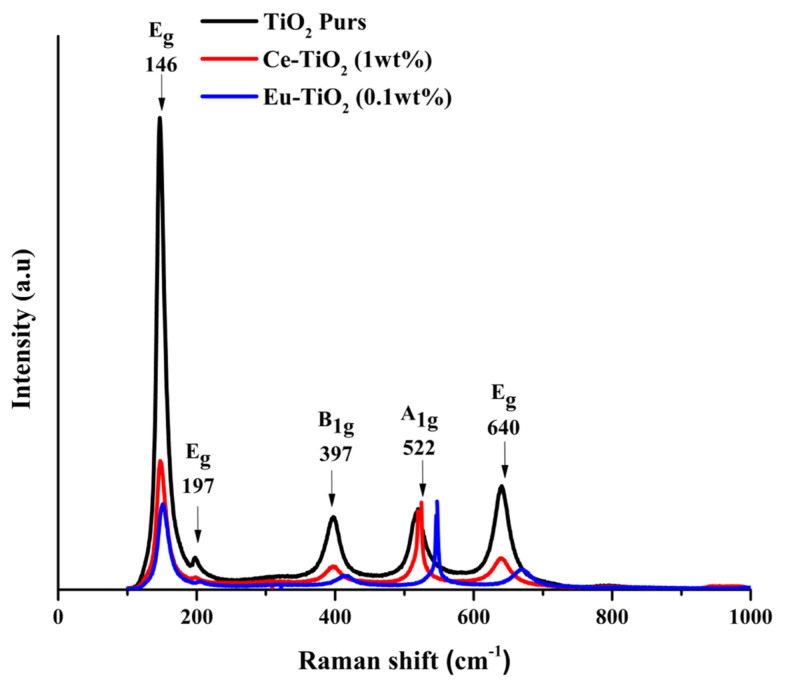
Raman spectra of pure- TiO_2_, Ce-doped TiO_2_ (1wt%), and Eu-doped TiO_2_ (0.1wt%) thin films annealed at 500 °C for 2h.

Figure 4 illustrates the Raman patterns of pure-TiO_2_, Ce-TiO_2_ (1wt%), and Eu-TiO_2_ (0.1wt%) thin films annealed at 500 °C for 2 h. It can be observed that there are five characteristics of active bands for the Raman spectrum of anatase which are (A_1g_ + 2B_1g_ + 3E_g_). Hence, Raman bands appeared at 146 (E_g_), 197 (E_g_), 397 (B_1g_), 522 (B_1g_), and 640 (E_g_) cm^−1^ are very distinctive and can be assigned to anatase phase, for all deposited thin films (Figure 4). Accordingly, it has been reported that in the Raman spectrum of anatase single crystal, the allowed modes appeared at 144 (E_g_), 197 (E_g_), 399 (B_1g_), 516 (A_1g_), 519 (B_1g_), and 639 cm^−1^ (E_g_) [54,55]. This finding is in accordance with XRD spectra analysis (Figure 3). In fact, Eg peak appears due to O–Ti–O symmetric stretching vibration in TiO_2_, B_1g_ peak appears due to O–Ti–O symmetric bending vibration and A_1g_ peak appears due to O–Ti–O antisymmetric bending vibration [55,56]. Raman spectra showed also distinct trends in the peak position of E_g_ mode with Ce and Eu doping to higher wavenumbers, respectively from 146cm^−1^ to 152cm^−1^ for 5wt% Ce-TiO_2_ and to 147cm^−1^ for 1wt% Eu-TiO_2_. The ionic size of Ce^3+^(0.111 nm), Ce^4+^(0.101 nm), Eu^3+^ (0.095 nm), and Eu^2+^ (0.109 nm) are larger than Ti^4+^(0.068 nm) ionic size, hence, doping TiO_2_ with Ce and Eu ions will distort the lattice structure of TiO_2_ and generates oxygen vacancies [56]. Oxygen vacancies generated are responsible for the shifting of the first Eg Raman peak [7]. Besides, the diminishing of Eg intensity after doping may be due to the breakdown of long-range translational crystal symmetry caused by the incorporated defects of Ce and Eu dopant ions [57].

### 3.4. Photo-electrochemical characterization

#### 3.4.1. Photopotential measurements

**Figure 5:**
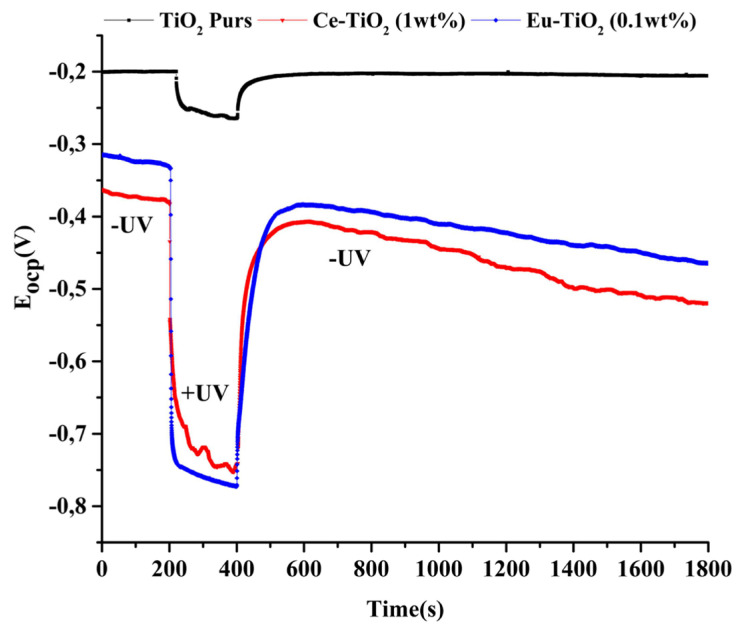
Open-circuit photopotential of Pure TiO_2_, Ce-TiO_2_ (1wt%), and Eu-TiO_2_ (0.1wt%) electrodes during UV On/Off cycle.

The response of photopotential measurements (Eocp) in an alkaline medium (NaOH 0.1M) during on/off cycle of UV irradiation is illustrated in Figure 5. During UV irradiation, the open circuit potential shifted towards more negative values for pure TiO_2_, Ce-TiO_2_ (1wt%), and Eu-TiO_2_ (0.1wt%) electrodes. The more cathodic values of Eocp indicated that more electrons were generated in the conduction band of the Ce-TiO_2_ (1wt%) and Eu-TiO_2_ (0.1wt%) photocatalysts, thus, the photogenerated charge carriers were successfully separated [34]. On the contrary, an exponential behavior appeared by cutting UV irradiation. The slow relaxation indicated a long lifetime of the electrons and holes for the Ce-TiO_2_ (1wt%) and Eu-TiO_2_ (0.1wt%) compared to pure TiO_2_ photocatalyst and an expected better photocatalytic activity during photodegradation reaction.

#### 3.4.2. EIS measurements p-Si and TiO_2_/ p-Si diagrams

**Figure 6:**
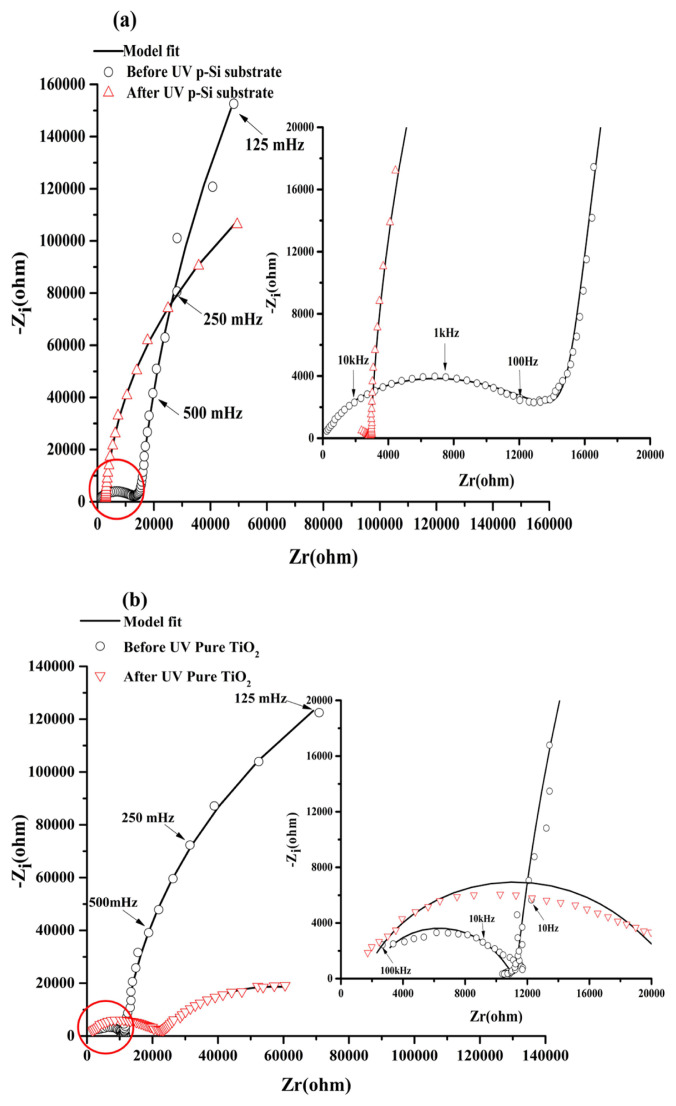
EIS in Nyquist representation (a) for p-Si and (b) Pure TiO_2_ electrodes in the dark before UV irradiation and after 2h of UV irradiation registered at open circuit potential in NaOH (0.1M).

**Figure 7:**
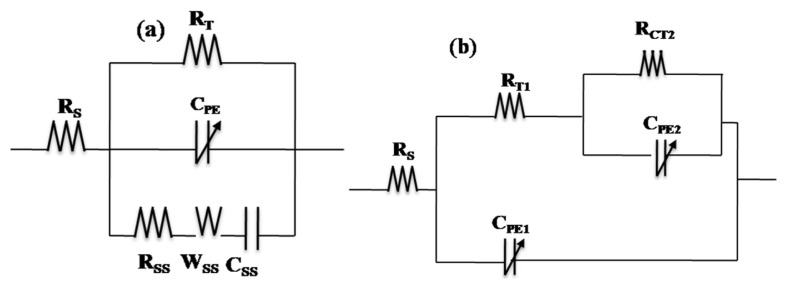
Equivalent circuit in the dark (a) before UV (b) after UV illumination.

In the dark before UV illumination, the Nyquist diagram of p-Si substrate (Figure 6a) presented two responses at high and low frequency ranges. It started by a semicircle at the high frequency range, then an almost vertical line of the curve, presenting a highly capacitive behavior (Figure 6a) characteristic of the semiconductor oxides. The same behavior is observed when the TiO_2_ film is fixed on the Si substrate. However, the high frequency loop presented a lower resistance and the highly capacitive behavior is attenuated (Figure 6b). This EIS behavior in the dark was represented by a circuit in the low frequency range (R_T_-C_PE_), that describes the behavior of the space charge layer of the semiconducting oxide (Figure 7a), where C_PE_ is used instead of pure capacitance. On the other hand, the second (R_SS_C_SS_W_SS_) circuit in the high frequency range describes the relaxation of charges via surface states (Figure 7a), where R_SS_, C_SS,_ and W_SS_ are respectively associated with the resistance, capacitance of surface states and Warburg element used to obtain a better fit. Both components of the equivalent circuit models are connected in parallel and the resulting circuit is connected in series with the electrolyte resistance (Rs) (Figure 7a). This proposed model was applied in previous work [58,34,59]. Table 3 presents the electrical components of the equivalent circuits before and after UV irradiation after fitting the experimental data of EIS diagrams using the software ZSimpWin3.2 with fitting error between E-3 and E-4. It was confirmed that the highly capacitive behavior (n = 0.95) decreased slightly after fixation of the TiO_2_ film (n = 0.94), however, the R_T_ decreased strongly from 1.17E6 to 4.036E5 (Ω) as observed by comparing the two Figures (6a and 6b). The presence of TiO_2_ at the p-Si surface decreased the space charge layer of the electrode. In the case of UV illumination for 2h and back to the dark for 1h, a set of new impedance diagrams was performed to evaluate the long-lasting photo-induced changes in the synthesized thin films. The experimental EIS plot of p-Si and TiO_2_ film on p-Si, showed the two time constant loops at the high and low frequencies. However, the arc radius at low frequency decreased in the two cases (Figures 6a and 6b), indicating that two electrodes possessed a lower electron transfer resistance after UV irradiation which leads to a faster interfacial charge transfer, thereby an effective separation of photogenerated electron/hole pairs occurred. Taking into accounts this charge transfer corresponding to the transfer of photogenerated holes to species in solution or present at the surface of the TiO_2_ semiconductor, the corresponding equivalent circuit model proposed can be described using two R_T_-C_PE_ circuits (Figure 7b). The small semicircle at high frequency range corresponds to the resistance for the charge transfer from p-Si through TiO_2_ to the surface (R_T1_, C_PE1_), whereas the large semicircle at low frequency range corresponds to the photoelectrode/electrolyte interfacial charge transfer resistance (R_CT2_, C_PE2_). Similar models have been adopted by other work [60]. The parameters of the equivalent circuit are summarized in the same Table 3.

##### Doped Ce or Eu-TiO_2_/p-Si diagrams Ce-TiO_2_/p-Si electrode

**Figure 8:**
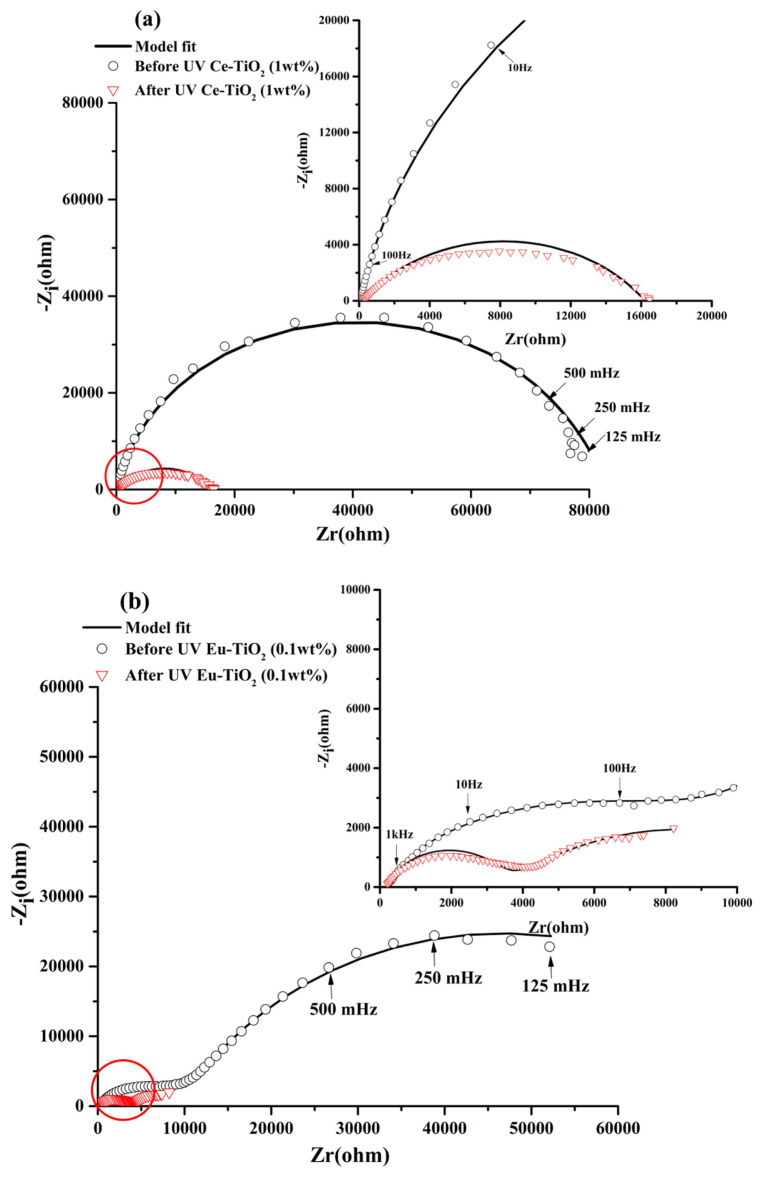
EIS in Nyquist representation for (a) Ce-TiO_2_ (1wt%) and (b) Eu-TiO_2_(0.1wt%) electrodes in the dark before UV irradiation and in the dark after 2h of UV irradiation registered at open circuit potential in NaOH (0.1M).

In the dark before UV illumination, the Nyquist diagrams of Ce-TiO_2_ (Figure 8a) did not present separation of the two observed loops. The related time constants seem to be in the same range or the time constant of the high frequency loop increased strongly from τ=210^−3^ms for non doped TiO_2_ to τ = 1.27ms due to the doping. The low frequency limit is near to 80000 (Ω) (Figure 8a) very low compared to the non doped electrode (Figure 6b). The Ce doping improved strongly the charge transfer in the dark. The R_T_ decreased to 8.26E4 (Ω) compared to the non doped electrode (4.036E5)(Ω) (Table 3). In the dark after UV illumination, the interface has the same behavior but the limit of the low frequency decrease drastically near to 16000(Ω) indicating that after UV irradiation the Ce-TiO_2_ electrode possessed a lower electron transfer resistance which leads to a faster interfacial charge transfer, which can lead to effective separation of photogenerated electron/hole (Figure 8a). Besides, the charge transfer resistance R_CT2_ decreased from 8.26E4 to 1.6E4(Ω) in comparison with the non doped electrode 7.34E4 (Ω) after UV illumination.

##### Eu-TiO_2_/ p-Si electrode

In the dark before UV illumination, the Nyquist diagrams of Eu-TiO_2_ (0.1wt%) showed a similar behavior compared to the non doped electrode with two separated loops but without a strong capacitive component (Figure 8b). The arc radius is lower, suggesting also an improved charge transfer due to the Eu doping. After UV illumination, the decrease is stronger and the Eu- doped electrode presented the lowest electron transfer resistance. In fact, the charge transfer resistance R_CT2_ decreased from 8.01E4 to 1E4 (Ω) compared to the non doped electrode (7.34E4 Ω after UV illumination). High carrier flow from the electrode to the interface with the solution is expected to lead to high separation between the photogenerated holes and electron and potentially high efficiency of the photocatalytic process when using this newly developed photocatalyst.

### 3.5. Photodegradation experiments

**Figure 9:**
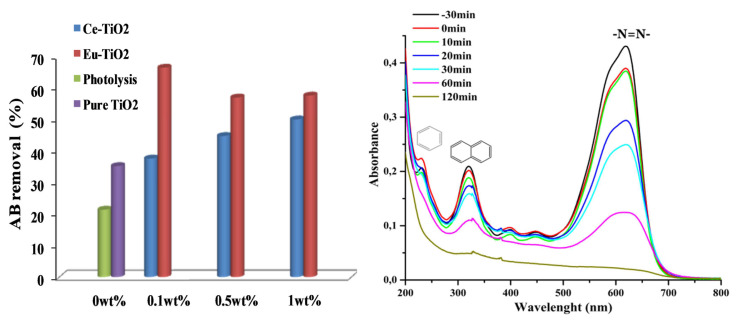
(a) AB removal (%) after 30 min of photodegradation by of Ce and Eu-TiO_2_ thin films (b) UV-Vis spectra of amido black withdrawn at different interval times for pure TiO_2_.

**Figure 10:**
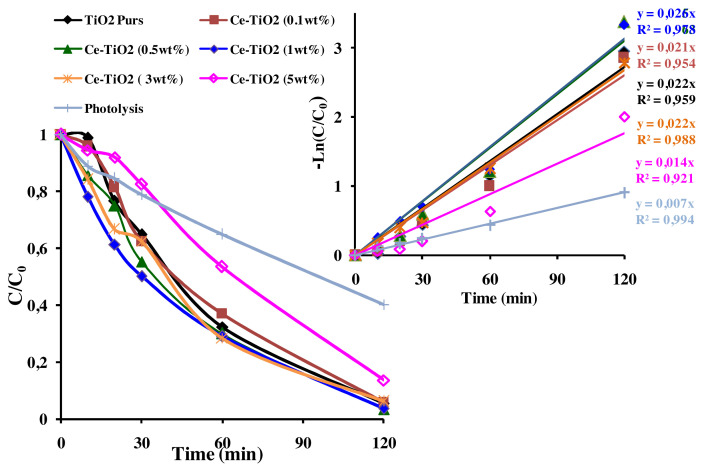
(a) C/C_0_ plot (b) Apparent 1^st^ order kinetics plot of AB (C_0_ = 10 ppm; pH= 3.5; λ = 620nm) for Ce-TiO_2_ thin films.

**Figure 11:**
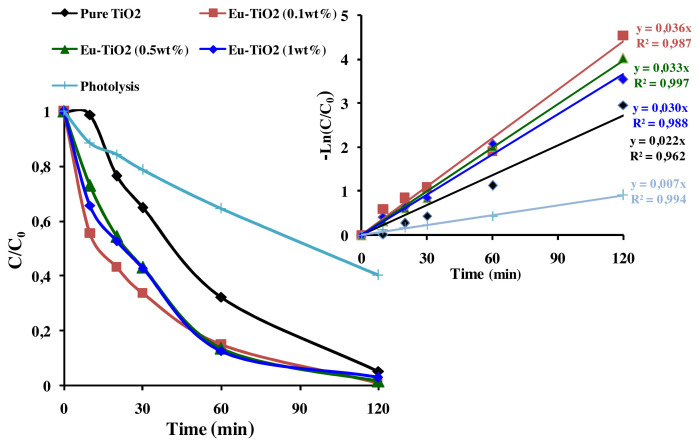
(a) C/ C_0_ plot (b) Apparent 1^st^ order kinetics plot of AB (C_0_ = 10ppm; pH=3.5; λ = 620nm) for Eu-TiO_2_ thin films.

The photocatalytic activity of pure Ce-TiO_2_ and Eu-doped TiO_2_ thin films was tested by investigating the photodegradation of AB under UV illumination. The obtained photodegradation efficiencies of AB (C_0_ = 10ppm, pH = 3.5) within 30 min, were summarized in Figure 9a. As shown in Figure 9a, doping TiO_2_ thin films by Eu ions increased the photodegradation efficiency after 30 min from 35.2% to 60%–66.4% depending on Eu amount (0.1, 0.5, and 1wt%). However, on Ce-doping, the photodegradation efficiency was enhanced from 35.2% to 50% after 30 min at optimal Ce amount (1wt%). Therefore, the best performance in AB removal was observed in the presence of Eu-ions dopant at an optimal concentration of 0.1wt%. Besides, the efficiencies were about 98.94%, 98.21%, and 97.13%, respectively for (Eu: 0.1, 0.5, and 1wt%) after 120 min of reaction time. It is worth noting that, the AB removal by photolysis reaction was about 60.52% after 120 min of reaction, which confirms the enhancement role of the developed photocatalysts, pure and Ce and Eu-TiO_2_ thin films. The UV-Vis spectra of AB are characterized by a remarkable peak at 620 nm and two ultraviolet peaks at 226 and 318 nm (Figure 9b). The peak at 620 nm is related to the presence of the azo chromophore group (N=N) (Figure 9b) [59]. While the peaks observed at 226 and 318nm (Figure 9b) are related respectively to the aromatic benzene and naphthalene groups [62,63]. After 120 min of photodegradation by pure TiO_2_ thin films, the aqueous solution of AB became totally colorless. The disappearance of the peaks at 226 nm and 318 nm (Figure 9b), indicates the degradation of AB under the experimental conditions (pH = 3.5, C_0_ = 10ppm). However, the intense peak at 620 nm decreased faster (35.19%) than the ultraviolet peak at 318 nm (24.28%) after 30 min (Figure 9b). It means that during the degradation mechanism, •OH radicals attack preferentially –N = N– group than the benzene and naphthalene groups and confirms the results of other works related to azo dyes [64]. The linear fit under optimal conditions of pH = 3.5 and C_0_ = 10 ppm can be approximated as apparent-first-order kinetics reaction. The apparent first-order kinetics of AB photodegradation are illustrated in Figures (10b and 11b) according to Eq.1.


(Eq.1) ln CC0=-kapp×t

where, t and *k_app_* are respectively the photodegradation time (min) and the apparent rate constant (min^−1^).

The parameters *k_app_* (min^−1^) and R^2^ (correlation coefficient) are mentioned in Figures (10b and 11b). After Ce-doping, *k_app_* of AB photodegradation slightly increased from *k_app_*= 0.022 min^−1^ for pure TiO_2_ to *k_app_*= 0.026 min^−1^ at optimal Ce amount (1wt%) corresponding to 96.44% of AB removal. But, it greatly increased from *k_app_*=0.022 min^−1^ for pure TiO_2_ to *k_app_*= 0.036 min^−1^ at optimal Eu amount (0.1wt%) reaching 98.94% of AB removal. Besides, the removal of AB was faster using Eu-doped TiO_2_ photocatalysts (t_1/2_ = 2min) than that of pure TiO_2_ (t_1/2_ = 45 min) and Ce-TiO_2_ (1wt%) (t_1/2_ = 30 min). However, it was clearly shown in Figures 10 and 11 that increasing the amount of Ce and Eu ions above an optimal amount (1wt% Ce) and (0.1wt% Eu), led to the decrease in *k_app_* to *k_app_* = 0.030 min^−1^ and *k_app_* = 0.014 min^−1^, respectively at Eu (1wt%) and Ce (5wt%). These results could be due to the presence of surface oxygen vacancies and Ti^3+^ surface states on the pure TiO_2_ surface that are not high enough to trap all the exciting photogenerated electron/hole pairs. Thereby, fewer amount of electrons and holes pairs are available to interact with the AB molecule [52]. Thus, adding appropriate amount of Ce and Eu ions led to better electron scavenging capacity due to the ability of unoccupied 4f orbital of Ce and Eu ions to accept electrons [65,66]. As a matter of fact, the electrons trapped in Ce^4+^/Ce^3+^and Eu^3+/^Eu^2+^ sites were subsequently transferred to the adsorbed O_2_ and produced superoxide radicals according to Equations (2, 3, 4, 5, and 6) and regenerate the dopants. Finally, oxygen radicals react with the protons in the aqueous solution to produce hydroxyl radicals (•OH) according to Eq. (7). Consequently, the photogenerated electron was transferred efficiently [8,67].


(Eq.2) $${\text{TiO}}_{2}+\text{h}\nu \to {\text{e}}^{-}+{\text{h}}^{+}$$

(Eq.3) $${\text{Ce}}^{4+}+{\text{e}}^{-}\to {\text{Ce}}^{3+}$$

(Eq.4) $${\text{Ce}}^{3+}+{\text{O}}_{2}\to {\text{Ce}}^{4+}+{\text{O}}_{2}^{•-}$$

(Eq. 5) $${\text{Eu}}^{3+}+{\text{e}}^{-}\to {\text{Eu}}^{2+}$$

(Eq. 6) $${\text{Eu}}^{2+}+{\text{O}}_{2}\to {\text{Eu}}^{3+}+{\text{O}}_{2}^{•-}$$

(Eq.7) $$O_{2}^{-}+2H^{+}\to 2{\text{OH}}^{•-}$$

However, according to Figures 10 and 11, the decrease in the photodegradation efficiency and *k_app_* when Ce and Eu amounts exceed respectively, 1wt% and 0.1wt% could be due to the reduction of the active sites on TiO_2_ surface covered by the excess amount of Ce and Eu ions [11]. On the other hand, when Ce and Eu dopant ions exceeded an optimal concentration, the thickness of the space charge layer (W) narrowed compared to the penetration depth of UV light. Thereby, increasing the recombination rate of electron/ hole pairs [52]. In summary, according to the present results, it was found that doping TiO_2_ thin films with Eu dopant ions greatly increase the photodegradation efficiency of AB than Ce dopant ions. The best photocatalyst (Eu-TiO_2_ 0.1wt%) will be considered in the following of this study for an optimization of the photocatalytic process.

#### 3.5.1. Effect of pH

**Figure 12:**
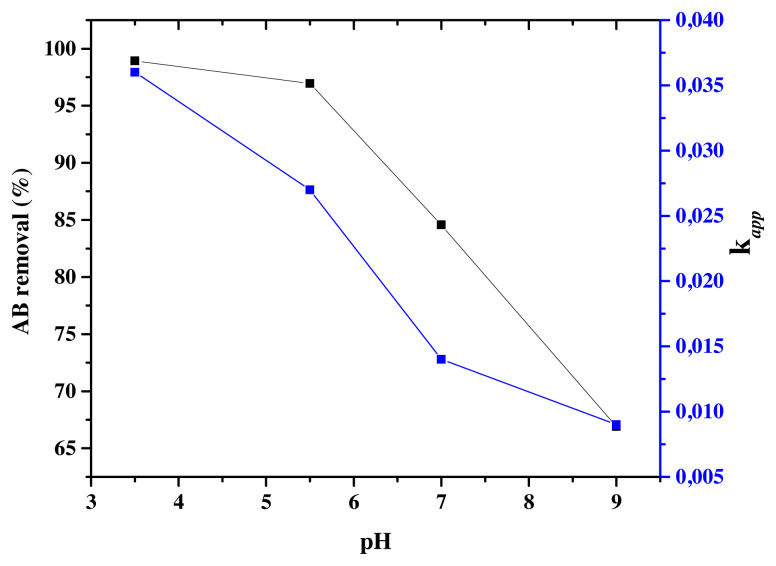
AB removal (%) and k*_app_* (min^−1^) for Eu-TiO_2_ (0.1wt%) thin films at C_0_ = 10 ppm; λ = 620 nm and different pH.

To study of the effect of pH in aqueous medium, the photocatalysts presenting the best photocatalytic activity Eu-TiO_2_ (0.1wt%) was considered. The photodegradation efficiencies of AB were studied at an initial pH range between 3.5 and 9 and C_0_ = 10ppm. It was found that the initial pH greatly affected the AB removal since it decreased from 98.94% to 66.90% (Figure 12), respectively when pH increased from 3.5 to 9. The parameters *k_app_* and R^2^ are mentioned in Figure 12 and Table 4. A decrease in *k_app_* of AB photodegradation was shown from *k_app_*=0.036 min^−1^ to *k_app_*=0.009 min^−1^, respectively for pH (3.5) to pH (9) (Table 4). As the TiO_2_ has an amphoteric behavior; the variation of pH solution changes its surface charge and shifts the catalytic reactions potentials [68,69]. In fact, the surface of TiO_2_ photocatalyst can be protonated in acidic media (Eq.8) or deprotonated in alkaline media (Eq.9).


(Eq.8) $$\text{TiOH}+{\text{H}}^{+}\to {\text{TiOH}}_{2}^{+}\;\text{pH}<pzc$$

(Eq.9) $$\text{TiOH}+{\text{OH}}^{-}\to {\text{TiO}}^{-}+{\text{H}}_{2}\text{O pH}>\text{pzc}$$
[70]

TiO_2_ has a pHpzc value of 6.25 which causes TiO_2_ to be positively charged at pH below pHpzc and to be negatively charged at pH above pHpzc. The lack of information about the pKa of AB dye, makes the prediction of its structure (protonated or ionic), which depends on the pH of the solution, more complex [71]. However, according to the present results, the optimum pH obtained is in the acidic range pH (3.5), where the Eu-TiO_2_ (0.1wt%) photocatalyst is positively charged, leading to the prediction of the AB molecule to be negatively charged. Thus, resulting in a favorable attraction between the Eu-TiO_2_ (0.1wt%) surface and the AB dye in the acidic solution and columbic repulsion in the alkaline solution [69,72]. Moreover, at lower pH, holes act as major oxidation species and at neutral or high pH, hydroxyl radicals are considered as the major oxidation species [73]. Therefore, under alkaline conditions the presence of hydroxyl ions may neutralize the acidic end-products produced by the photodegradation reaction, thus leading to a decrease in the photodegradation of amido black [74]. As a consequence, the photodegradation efficiency of AB under acidic medium is enhanced by the positive holes which are considered as the major oxidation species at low pH and the strong adsorption of the anionic dye molecules on the positive surface of the photocatalyst [75].

#### 3.5.2. Effect of dye concentration

**Figure 13:**
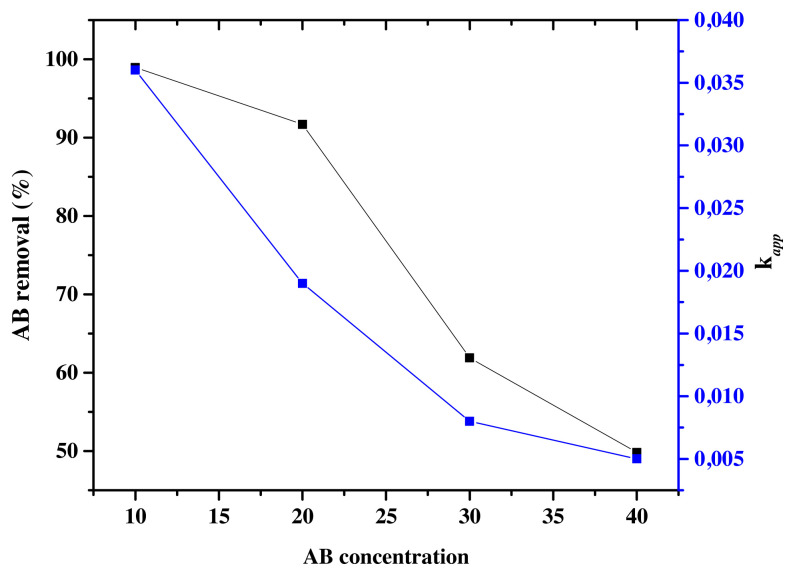
AB removal (%) and k*_app_* (min^−1^) for Eu-TiO_2_ (0.1wt%) thin films at pH = 3.5; λ = 620 nm and different AB concentrations.

The initial dye concentration is an important factor that affects the photocatalytic degradation of AB. The effect of initial dye concentration was studied by varying the initial concentration to (C_0_ = 10,20,30 and 40 ppm) at constant pH = 3.5 under optimal conditions of atomic weight percentage of Eu (0.1wt%). According to Figure 13, the reaction rates of AB photodegradation decrease from *k_app_*= 0.036 min^−1^ for C_0_ = 10ppm to *k_app_* = 0.005 min^−1^ for C_0_ = 40ppm in accordance with the decrease in the photodegradation efficiency (Table 4). In fact, the photodegradation efficiency of AB decreased from 98.94% to 49.85%, respectively when C_0_ increased from 10 ppm to 40 ppm. Similar results have been reported for the photodegradation of AB dye [63]. The more the initial dye concentration increases, the more organic substances are adsorbed on the surface of TiO_2_, whereas less number of photons are available to reach the catalyst surface, and therefore less •OH are formed, thus resulting in the decrease of the degradation efficiency. The penetration of light to the surface of the catalyst is the limiting step [69,74]. On another side, the high coloration of the solution due to the high concentration of the colorant prevents the catalyst from being excited and favorites the photolysis of AB.

#### 3.5.3. Degradation pathways and oxidation reaction intermediates of AB

**Figure 14:**
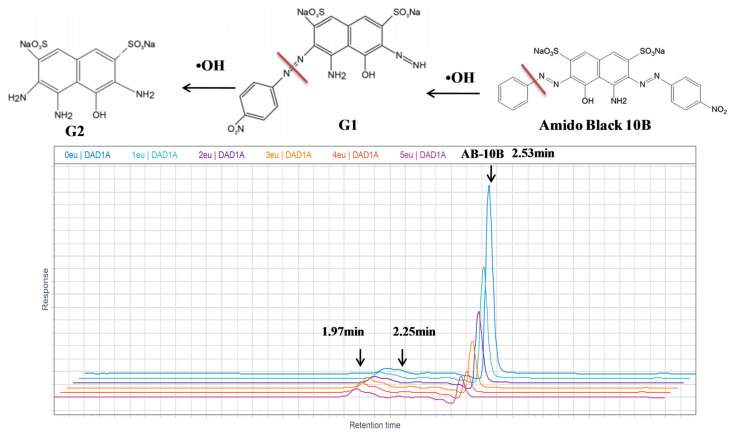
Proposed amido black 10B degradation pathway [61] and high-performance liquid chromatograms of AB obtained at different interval times of irradiation (pH = 3.5, C_0_ = 10 ppm), using Eu (0.1wt %) doped TiO_2_ films.

**Figure 15:**
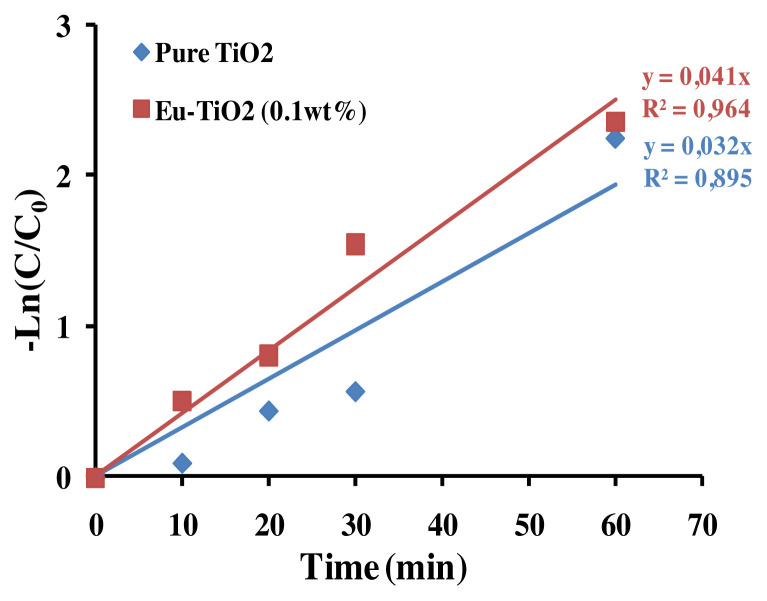
Photocatalytic mineralization of AB in aqueous solution (C_0_ = 10ppm, pH = 3.5) using Pure TiO_2_ and Eu-TiO_2_ (0.1wt%) photocatalyst.

The photodegradation of AB by Eu-TiO_2_ (0.1wt%) thin films, under optimal conditions (pH = 3.5, C_0_ = 10ppm), was investigated using HPLC and compared to pure TiO_2_ photocatalyst. The chromatograph shows that the intense peak at the retention time of 2.53 min refers to the AB dye molecule. After decolorization occurred, the chromatogram of the degraded dye showed a considerable decrease of the major peak (Figure 14). The disappearance of the AB dye peak was followed by a generation of new peaks at 2.25 min and 1.97 min retention time, in the HPLC chromatogram of AB photodegradation reaction (Figure 14). Thus, we can confirm the degradation of the initial AB dye molecule and its transformation into intermediates. Based on the mechanisms of amido black degradation proposed by [63], it was reported that the degradation of AB is initiated by radical attack on preferential sites of the molecules, the nitro, sulfonate, and azo groups. The degradation pathway can be complex and can lead to the generation of a multitude of intermediates. 4-amino-6-diazenyl-5-hydroxy-3-((4-nitrophenyl)diazenyl)naphthalene-2,7-disulfonate (G1) is considered as a major intermediate [63]. Then the attack of •OH radicals produced sodium 3,4,6-triamino-5-hydroxynaphthalene-2,7-disulfonate (G2) (Figure 14). According to current investigation, the apparition of two intermediates peaks at 1.97 min and 2.25 min, respectively after 10 min and 30 min of starting the photodegradation reaction, could be correlated to the fragment (G1) and (G2) detected by [63] after photodegradation of amido black 10B by Polycarbazole-Decorated TiO_2_ Nanohybrids (Figure 14). Furthermore, the apparent-first constant of AB degradation using Eu-TiO_2_ (0.1wt%) photocatalyst under 60 min of photodegradation reaction was about *k_app_*= 0.041min^−1^ compared to *k_app_*= 0.032min^−1^ for pure TiO_2_ (Figure 15). This confirms that doping TiO_2_ thin films with Eu ions with an appropriate amount (0.1wt%) leads to an enhancement of mineralization efficiency of AB dye obtained by HPLC results, which is in accordance with UV-Vis results that showed an enhancement of AB discoloration by the Eu-TiO_2_(0.1wt%) photocatalyst. In summary, the degradation mechanism can be assumed to be the •OH radicals attack of the azo groups of AB molecule [76].

## 4. Conclusion

In summary, Ce and Eu-TiO_2_ thin films were synthesized successfully by sol-gel dip-coating technique and coated on silicon wafers by a sol precursor solution containing different ratio of Ce and Eu ions. EIS measurements revealed that the doped TiO_2_ photocatalyst by Ce and Eu ions presented a faster interfacial charge transfer and an expected higher photocatalytic activity during the photoreaction, which was confirmed by the photodegradation results. According to the present study, doping TiO_2_ thin films with Eu ions can effectively enhance the photodegradation and the mineralization efficiency of AB removal at an optimal concentration of Eu (0.1wt%) for better electron/hole pairs separation. The reaction rate constant *k_app_* of AB removal by Eu-TiO_2_ (0.1wt%) was about *k_app_=* 0.036 min^−1^ and t_1/2_ was around 12 min compared to (t_1/2_ = 45 min, t_1/2_ = 30 min) and (*k_app_* = 0.022 min^−1^, *k_app_ =* 0.026 min^−1^), respectively for Pure TiO_2_ and Ce-TiO_2_ (1wt%) photocatalysts.

The study also revealed that the initial pH and AB concentration greatly affected the photodegradation of AB. The maximum photodegradation efficiency reached 98.94% in acidic media at pH = 3.5 and C_0_ = 10ppm.

## Figures and Tables

**Table 1:** Effect of different Ce and Eu content on thickness of the prepared TiO_2_ doped thin films, and atomic ratio Ce/Ti and Eu/Ti.

Sample	Thickness (μm)	Ce/Ti and Eu/Ti atomic ratio
Pure TiO_2_	6.4	0
0.1wt% Ce-TiO_2_	4.5	0.1
0.5wt% Ce-TiO_2_	4.1	0.5
1wt%Ce-TiO_2_	2.7	0.8
3wt%Ce-TiO_2_	1.4	1.3
5wt%Ce-TiO_2_	3.7	2.2
0.1wt% Eu-TiO_2_	2.7	0.1
0.5wt% Eu-TiO_2_	1.4	0.1
1wt% Eu-TiO_2_	2.7	0.4

**Table 2:** Effect of different Ce and Eu content on calculated parameters of (101) crystal plane of the first anatase peak diffraction at 500 °C during 2h for Pure- TiO_2_, Ce-doped TiO_2_ and Eu-doped TiO_2_ thin films.

Sample	Position (2 Ɵ)	FWHM	d- spacing (nm)	Average cristalline size D (nm)
Pure TiO_2_	25.30	0.307	3.03	27.71
Ce-TiO_2_ (0.1 wt%)	25.24	0.307	3.03	27.69
Ce-TiO_2_ (0.5 wt%)	25.28	0.307	3.04	27.70
Ce-TiO_2_ (1 wt%)	25.28	0.307	3.03	27.70
Ce-TiO_2_ (3 wt%)	25.22	0.307	3.53	27.69
Ce-TiO_2_ (5 wt%)	25.20	0.409	3.53	20.88
Eu-TiO_2_ (0.1 wt%)	25.25	0.307	3.03	27.69
Eu-TiO_2_ (0.5 wt%)	25.27	0.307	3.03	27.69
Eu-TiO_2_ (1 wt%)	25.25	0.307	3.03	27.69

**Table 3:** Numerical results for the electrical components used in the equivalent circuits.

Before UV illumination	R_S_(Ω)	R_T_(Ω)	C_PE_(Ω^1^s^n^)	n	R_SS_(Ω)	C_SS_(μF)	W_SS_(μF)
**p-Si**	1.74E2	1.17E6	7.82E-6	9.5E-1	1.55E4	2.25E-9	7.7E-7
**PureTiO** **_2_**	2.21E3	4.036E5	8.01E-6	9.4E-1	9.28E3	5.26E-10	8.92E-8
**Ce-TiO** **_2_** ** (1wt%)**	57.92	8.26E4	1.28E-6	8.8E-1	4.29E4	1.08E-8	1.58^E^-6
**Eu-TiO** **_2_** ** (0.1wt%)**	2.63E2	8.01E4	1E-5	7.8E-1	1.29E4	9.66E-9	2.41E-6
**After UV illumination**	R_S_(Ω)	R_T1_(Ω)	C_PE1_(Ω^1^s^n^)	n_1_	R_CT2_(Ω)	C_PE2_(Ω^1^s^n^)	n_2_
**p-Si**	1.47E3	2.83E3	1.77E-8	6.4E-1	2.8E5	9.57E-6	9.74E-1
**Pure TiO** **_2_**	1.47E3	1.92E4	1.18E-8	8E-1	7.34E4	1.428E-5	6E-1
**Ce-TiO** **_2_** ** (1wt%)**	1.86E2	7E3	8.71E-7	6.2E-1	1.6E4	1.04E-3	9.9E-1
**Eu-TiO** **_2_** ** (0.1wt%)**	27E2	3E3	1.94E-7	8E-1	1E4	1.21E-4	4.7E-1

**Table 4:** Kinetic parameters of AB photodegradation by Ce and Eu-TiO_2_ under different pH and AB concentration.

Photocatalyst	pH	*C* *_0_* * ppm*	*k_app_* (min^−1^)	R^2^	pH	*C_0_* ppm	*k_app_* (min^−1^)	R^2^
**Eu-TiO** **_2_** ** (0.1wt%)**	3.5 5.5 7.0 9	10	0.036 0.027 0.014 0.009	0.987 0.983 0.980 0.968	3.5	10 20 30 40	0.036 0.019 0.008 0.005	0.987 0.982 0.974 0.996
**Ce-TiO** **_2_** ** (1wt%)**	3.5	10	0.026	0.978				
**Pure TiO** **_2_**	3.5	10	0.022	0.959				
**Photolysis**	3.5	10	0.007	0.994				
